# Towards lead-free perovskite photovoltaics and optoelectronics by *ab-initio* simulations

**DOI:** 10.1038/s41598-017-13172-y

**Published:** 2017-10-25

**Authors:** Md Roknuzzaman, Kostya (Ken) Ostrikov, Hongxia Wang, Aijun Du, Tuquabo Tesfamichael

**Affiliations:** School of Chemistry, Physics and Mechanical Engineering and Institute of Future Environments, Queensland University of Technology, QLD 4000 Brisbane, Australia

## Abstract

Lead (Pb) free non-toxic perovskite solar cells have become more important in the commercialization of the photovoltaic devices. In this study the structural, electronic, optical and mechanical properties of Pb-free inorganic metal halide cubic perovskites CsBX_3_ (B = Sn, Ge; X = I, Br, Cl) for perovskite solar cells are simulated using first-principles Density Functional Theory (DFT). These compounds are semiconductors with direct band gap energy and mechanically stable. Results suggest that the materials have high absorption coefficient, low reflectivity and high optical conductivity with potential application in solar cells and other optoelectronic energy devices. On the basis of the optical properties, one can expect that the Germanium (Ge) would be a better replacement of Pb as Ge containing compounds have higher optical absorption and optical conductivity than that of Pb containing compounds. A combinational analysis of the electronic, optical and mechanical properties of the compounds suggests that CsGeI_3_ based perovskite is the best Pb-free inorganic metal halide semiconductor for the solar cell application. However, the compound with solid solution of CsGe(I_0.7_Br_0.3_)_3_ is found to be mechanically more ductile than CsGeI_3_. This study will also guide to obtain Pb-free organic perovskites for optoelectronic devices.

## Introduction

Recently, metal halide perovskites have drawn a great attention in the scientific community^1–11^ due to their remarkable performance in solar cells thanks to their outstanding opto-electronic properties such as tunable bandgap, high optical absorption, broad absorption spectrum, small carrier effective masses, dominant point defect, long charge diffusion lengths and high charge carrier mobility^1,3^. In addition to this, these materials are abundant in nature and inexpensive. As a result solar cells based on these materials would be cheaper and more efficient than silicon-based photovoltaic (PV) technology^1^. Because of these extraordinary properties, this group of semiconductors have the potential to be used in a wide range of electronic devices beyond solar cells such as light emitting diodes, photodetector and solar-to-fuel energy conversion devices^8–11^. To predict a specific device application and improvements, a deeper and fundamental understanding the properties of the semiconductors is necessary. Therefore, studying the electronic, optical and mechanical properties as well as understanding the overall characteristics of the system is utmost important.

Metal halide perovskite materials can be described by the general formula ABX_3_, where A is a cation, B is a metal ion and X is a halogen anion. In the last few years, different properties of metal halide perovskites have been reported for single or group of compounds (see Supplementary Section “Literature Review”) to be used in solar cells after the discovery of organic perovskites^12^. Most of the perovskite compounds with high performance contain lead (Pb) which is toxic and undesirable. Herein, we have considered Pb-free inorganic metal halide perovskite compounds to understand their general characteristics using first-principles Density Functional Theory (DFT). This work focuses on investigation of the structural, electronic, optical and mechanical properties of Pb-free metal halide perovskites CsBX_3_ (B = Sn, Ge; X = I, Br, Cl) and compared with Pb-containing compounds CsPbX_3_ (X = I, Br, Cl). The atoms Sn and Ge have been chosen to replace Pb because of their similarity in chemical composition and valency from the periodic table.

The investigation of the optical properties of solids is important for better understanding the electronic properties of the materials. The study of the optical functions also reveals the response of a material to light. Therefore, deep knowledge on the optical parameters is essential for the practical applications of a material in optoelectronic devices like solar cell, diode, laser, etc. However, less study has been carried out on the optical properties of the considered perovskite compounds. In particular the optical properties of CsGeX_3_ (X = I, Br, Cl) materials are still unexplored in detail. Also understanding the mechanical properties of these materials including mechanical stability, rigidity and ductility are useful to predict the importance of the materials for industrial applications. To the best of our knowledge, the elastic constants and moduli of the Pb-free CsSnI_3_, CsSnCl_3_, CsGeI_3_ and CsGeCl_3_ compounds are still not reported. Therefore, we have considered selected metal halide perovskite compounds and investigated their structural, electronic, optical and mechanical properties using first-principles Density Functional Theory (DFT) to obtain suitable Pb-free perovskites for solar cells and other optoelectronic devices.

## Results and Discussion

### Structural properties

**Figure 1:**
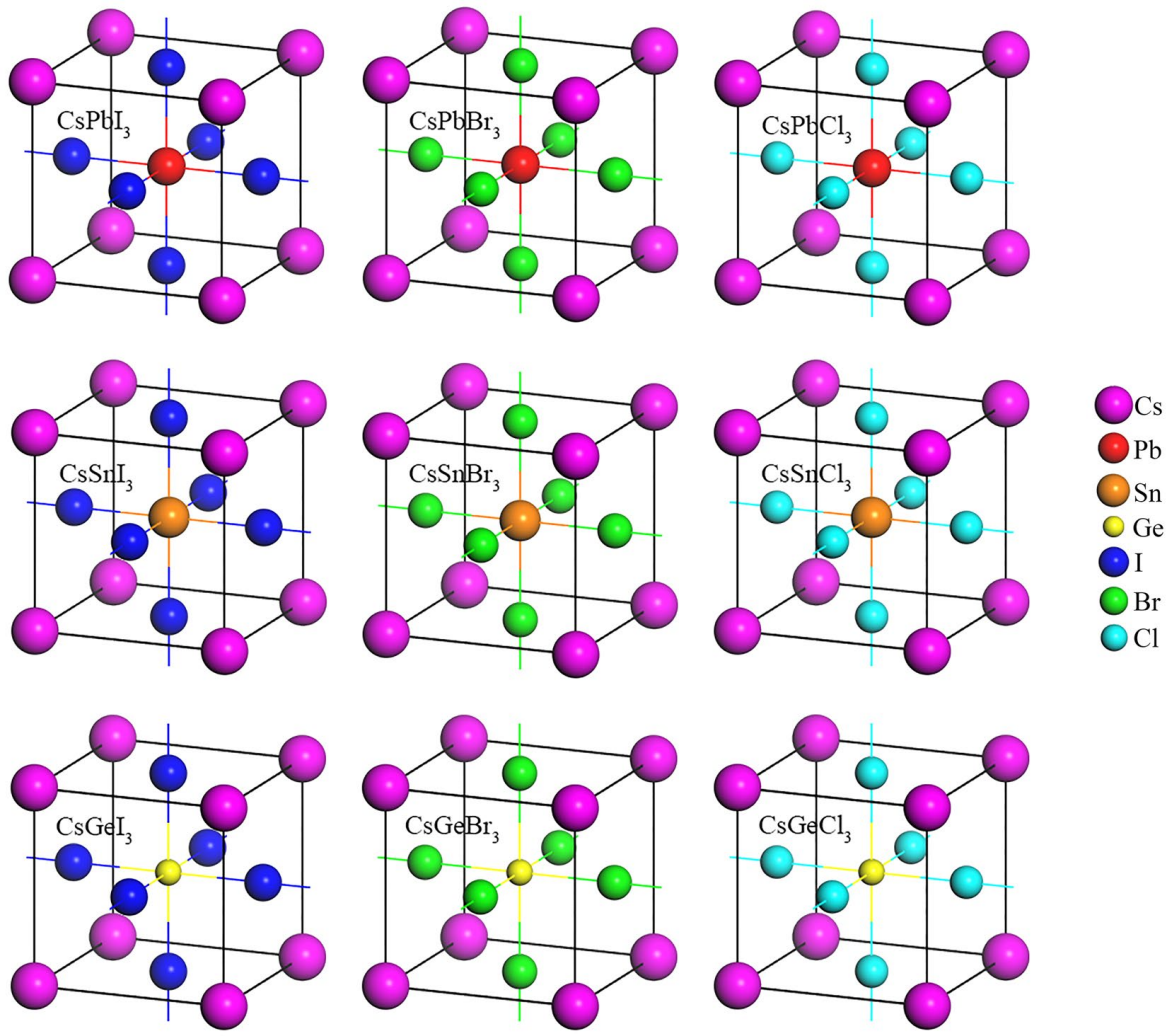
Unit cells of the considered cubic metal halide perovskites CsBX_3_ (B = Sn, Ge; X = I, Br, Cl) as compared with Pb-based compounds CsPbX_3_ (X = I, Br, Cl). Replacement of halogen atoms is showing from left to right while the replacement of Pb is showing from top to bottom. The molecular models are optimized by DFT calculations.

The cubic metal halide perovskites CsBX_3_ (B = Pb, Sn, Ge; X = I, Br, Cl) have space group ${Pm}\mathop{3}\limits^{\bar{} }m$ (no. 221). The unit cell of the material contains five atoms with one formula unit shown in Fig. 1. In the unit cell, the Cs atoms occupy the corner positions with 1*a* Wyckoff site and (0, 0, 0) fractional coordinates, the B atoms occupy the body centred position with 1*b* Wyckoff site and ($\frac{1}{2},\,\frac{1}{2},\,\frac{1}{2}$) fractional coordinates, and the X atoms occupy the face centred positions with 3*c* Wyckoff site and ($0,\,\frac{1}{2},\,\frac{1}{2}$) fractional coordinates.

**Figure 2:**
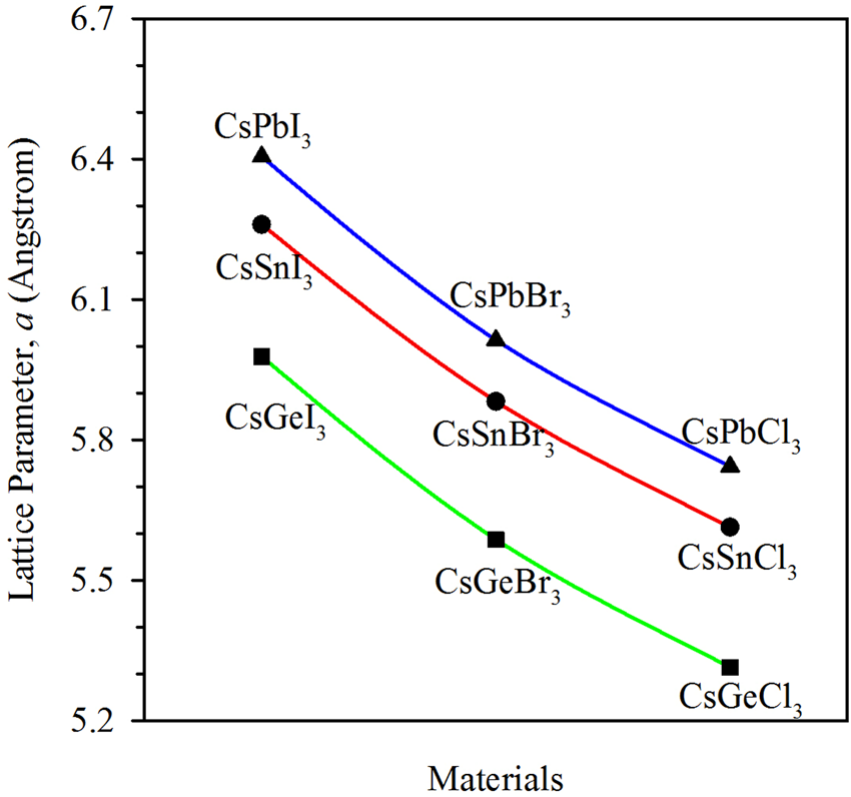
Variation of lattice parameter due to the replacement of atoms by similar atoms. The lattice parameter is seen to change periodically depending upon the size of atoms in the unit cell.

The calculated equilibrium lattice parameter *a* and the volume of the optimized cells are in good agreement with the experimental results as well as with the available theoretical results (see Supplementary Table 1). We have also found that the replacement of halogen atom (X) by a lighter and smaller halogen atom reduces the lattice parameter (Fig. 2) and the unit cell volume (Supplementary Figure 1). Similarly, the replacement of Pb by tin (Sn) or germanium (Ge) causes the reduction of lattice parameter of perovskites as shown in Fig. 2. A clean periodic behaviour in term of the change in lattice parameter as well as the unit cell volume is observed with the considered metal halide perovskite materials when changing the size of halides. This is a general features for the most compounds and it is because of the change in atomic size and charge.

The replacement of one type of atoms by a similar atom from a compound can affect the structural parameters of the compound. As a consequence, the material properties like electronic, optical and mechanical properties are also affected. Thus, one can expect to tune the properties of a material by replacing the constituent atoms with the atoms which are in similar groups in the periodic table.

### Electronic properties

**Figure 3:**
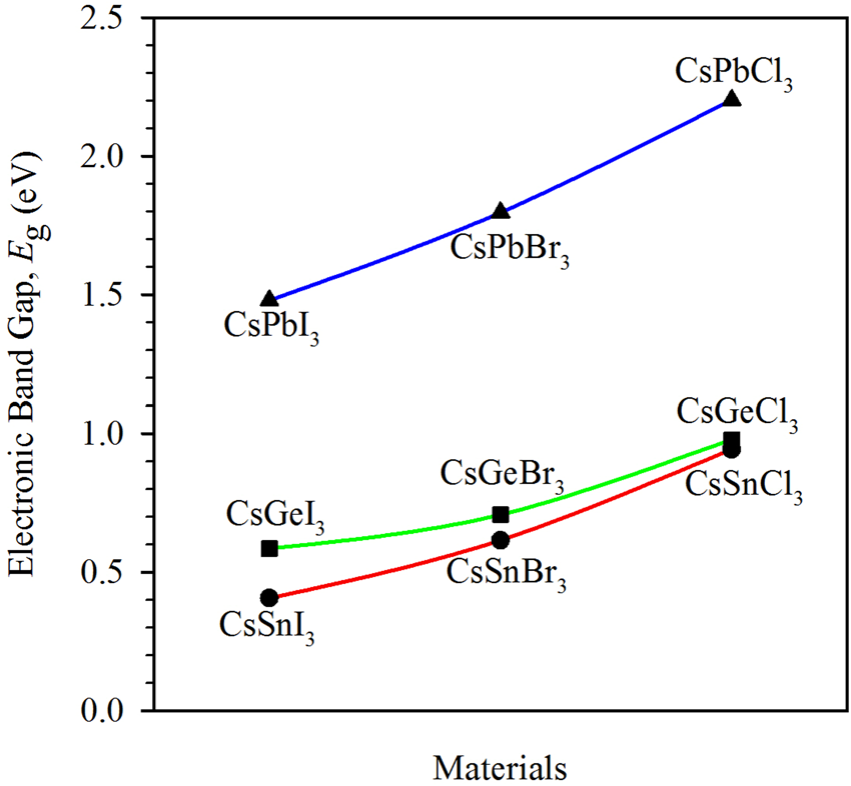
Variation of the electronic band gap due to the replacement of atoms by similar atoms.

We have calculated the electronic band gap using generalized gradient approximation (GGA) of Perdew-Berke-Ernzerhof (PBE). The calculated electronic band gap values are found to be in good agreement with the available results in the literature using the same theory (see Supplementary Table 2) while some diversity has been found with other results. Sometimes hybrid potential like HSE (Heyd-Scuseria-Ernzerhof)^13^ may give better estimation^14^. But it is not the perfect one as diversity has been found for some compounds (see Supplementary Table 2). Thus, it is still a challenge to find the appropriate potential to predict the theoretical electronic band gap for this system of materials. Similar to the unit cell volume, the electronic band gap is seen to change periodically for changing the halogen atoms as shown in Fig. 3. Here, higher band gap is observed for the compounds containing lighter halogen atoms. However, exception is observed in case of replacement of Pb by Sn and Ge. The Ge containing compounds have higher electronic band gaps than Sn containing compounds as for Ge, the electronic states of conduction band slightly shift towards the high energy and this is very common for this group of compounds^14^.

The study of the electronic band structure (Supplementary Figure 3) suggests that the pattern of the band structure is almost not affected by replacement of a halogen atom with other halogen atoms. Due to this characteristics, metal halide perovskites are suitable to be used in light emitting diodes (LED) as the band gap of these materials can easily be tuned by changing the halogen contents while the other properties remain almost same. Also, from the total and partial densities of states (Supplementary Figure 4) one can expect that the replacement of I by Br and Cl increases the number of electronic states in valence band just below the Fermi level. But the opposite trend is observed during the replacement of Pb by Sn or Ge. Moreover, the total DOS below the Fermi level mainly comes from the *p* orbital of halogen atoms. In addition to this the total DOS above the Fermi level is mainly contributed by *p* orbital of B atoms as from Pb-6*p*, Sn-5*p* and Ge-4*p*.

### Optical properties

The detail optical properties including real and imaginary part of dielectric functions, refractive index, absorption spectra, reflectivity and photoconductivity of our considered compounds are investigated up to the photon energy of 30 eV to reveal the response of the materials in solar and high energy radiation (see Supplementary Section “Optical Functions”).

**Figure 4:**
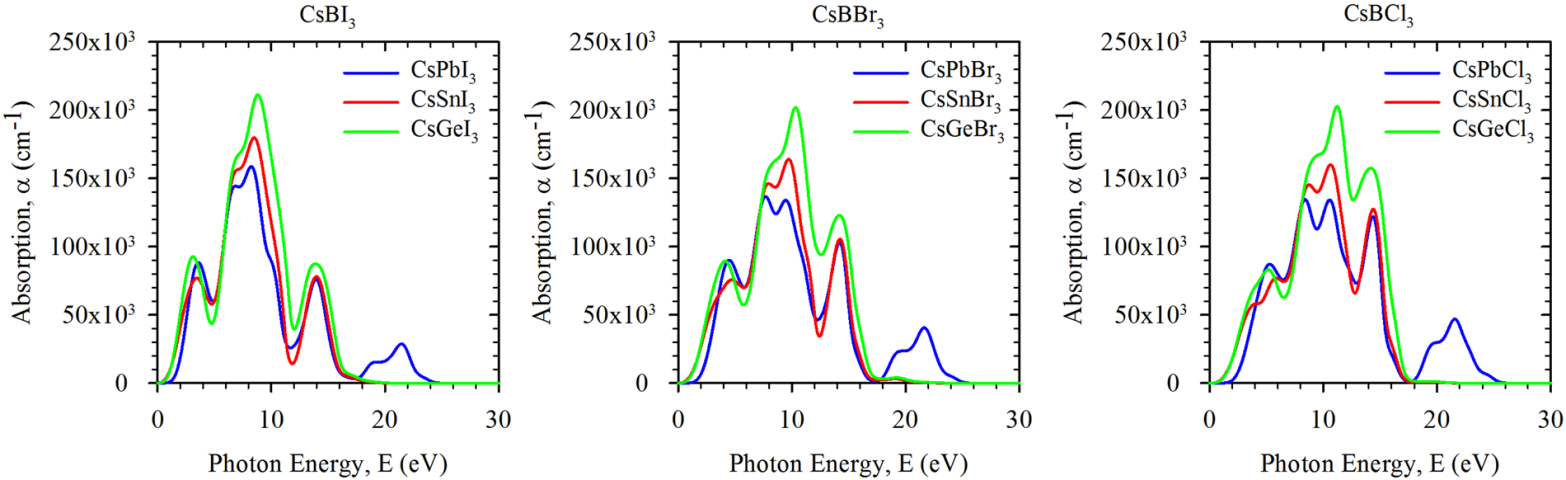
Calculated optical absorption of perovskites CsBX_3_ (B = Pb, Sn, Ge; X = I, Br, Cl) as a function of incident photon energies.

The optical absorption coefficient of a material is a measure of the penetration of light at specific wavelength (energy) into the material before it gets absorbed^15^. It also gives information about the optimum solar energy conversion efficiency which is important for the practical application of a material in solar cell. The calculated optical absorption spectra of the considered compounds under the simulated illumination is presented in Fig. 4. In general, there are mainly three light absorption peaks observed for all compounds except those containing Pb for which an additional peak is observed in high energy region. The replacement of Pb by Sn or Ge affects the magnitude of the absorption spectra, but the position of the peaks remain at the same energy. However, the replacement of I by Br and Br by Cl shift the position of the peaks towards the higher energy. Most of the considered compounds show high absorption between the energy ranges from 2–16 eV with first peak at about 4 eV, second broad peak centred at about 10 eV and third peak at about 14 eV. It is also found that the maximum absorption is obtained with Ge based compounds compared to Pb and Sn based counterpart. Therefore, Ge would be a better substitute of Pb in the inorganic perovskites for application in solar cells^10^. The efficiency of the Ge based inorganic perovskite solar cells (CsGeX_3_) is, however, to be investigated if it can be competitive with the existing organic-inorganic hybrid perovskite solar cells, such as (CH_3_NH_3_)PbX_3_.

**Figure 5:**
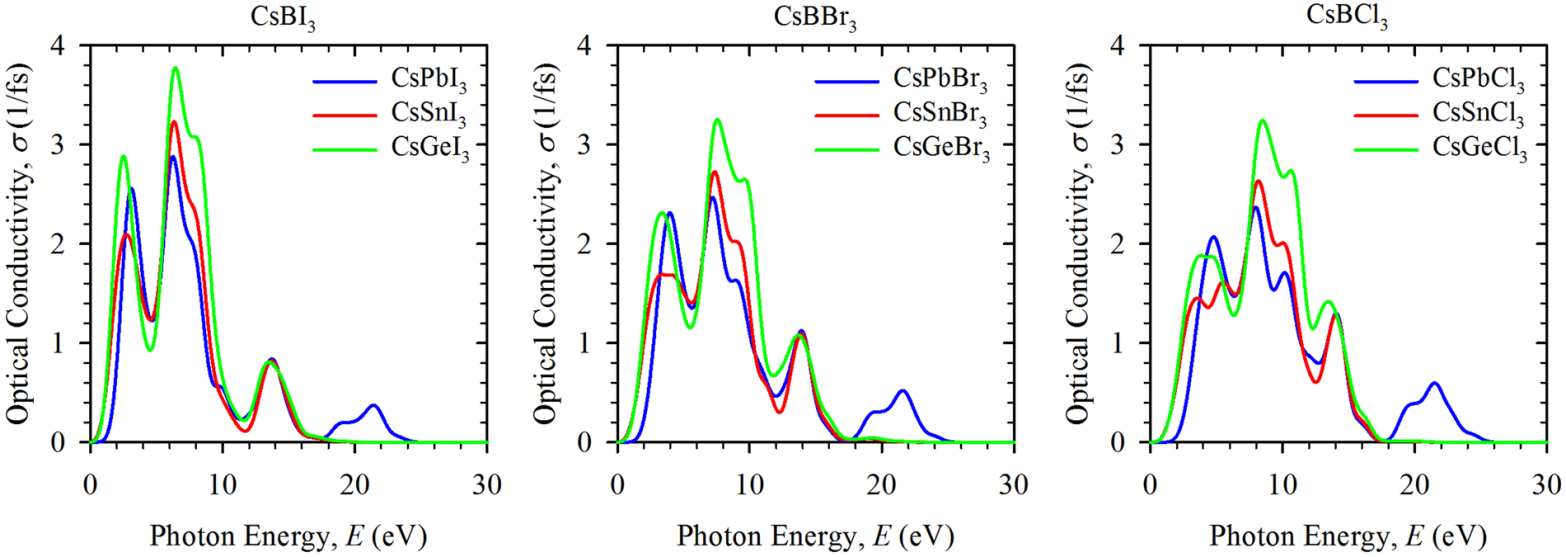
Calculated optical conductivity of perovskites CsBX_3_ (B = Pb, Sn, Ge; X = I, Br, Cl) as a function of incident photon energies.

Reflectivity is another important optical property for the solar cell and other applications of the perovskite. All the investigated perovskite materials show a low reflectivity in the IR-Visible-UV region of the spectra (see Supplementary Figure 7). This low reflectivity suggests that the materials have high absorptivity and/or transmitivity. However, our study of the dielectric function (see Supplementary Section “Optical Functions”) suggests that the materials have high transmitivity at high energy region and low transmittance in low energy region. Therefore, the results of the reflectivity and dielectric function confirm that the perovskite compounds have high absorption at low energy (<10 eV) region of the energy spectrum. On the other hand, the optical conductivity is a good reflection of the photoconductivity^16^. The real part of the optical conductivity is shown in Fig. 5. As can be seen, most of the perovskite compounds have high optical conductivity at low energy region. Similar to the phenomenon observed in absorption, Ge based compounds have higher optical conductivity than other metal cations based materials. Moreover, the magnitude of the spectra is higher for I containing compounds, while the spectra is seen to broaden for Br and Cl. In addition, the higher value of the extinction coefficient (Supplementary Figure 9) in the low energy region of the spectra also reveals that Ge is found to be a better replacement of Pb for solar cells.

### Mechanical properties

The elastic constants are essential to predict the mechanical stability of a crystal. The well-known Born stability criteria^17^ for cubic crystal is given by: ${C}_{11}-{C}_{12} > 0,\,{C}_{11}+2{C}_{12} > 0,\,{C}_{44} > 0$. The three independent elastic constants (*C*_11_, *C*_12_ and *C*_44_) of the cubic perovskite compounds are calculated using the finite strain theory^18^ implemented in CASTEP module and presented in Table 1 with available theoretical and experiment results. The calculated elastic constants for CsPbCl_3_ are in good agreement with the only available theoretical results reported in literature^19^. Also, the calculated results for CsSnBr_3_ and CsGeBr_3_ are in good agreement with the available theoretical results reported by Brik^20^. The calculated elastic constants are positive and satisfy the Born stability criteria, which implies that the compounds are mechanically stable.

**Table 1:** The calculated and the available theoretical and experimental values of elastic constants *C*_ij_ (GPa), bulk modulus *B* (GPa), shear modulus *G* (GPa), Young’s modulus *Y* (GPa), Pugh’s ratio *B*/*G* and Poisson ratio *υ* of cubic perovskites CsBX_3_ (B = Pb, Sn, Ge; X = I, Br, Cl).

Phase	*C*_11_	*C*_12_	*C*_44_	*B*	*G*	*Y*	*B*/*G*	*v*	References
CsPbI_3_	34.23	4.46	3.24	14.38	6.30	14.49	2.283	0.32	This Work
				23					Cal.^21^
				19.8					Cal.^22^
				14.4					Cal.^23^
					7.9	20.1	2.544	0.33	Exp.^24^
CsPbBr_3_	42.32	6.51	4.24	18.45	7.90	20.74	2.335	0.31	This Work
				27					Cal.^21^
				23.5					Cal.^22^
CsPbCl_3_	48.97	7.83	5.08	21.54	9.27	24.32	2.324	0.31	This Work
	46.04	6.10	4.91	22.59	8.98	23.34	2.16	0.30	Cal.^19^
				31					Cal.^21^
				25.8					Cal.^22^
CsSnI_3_	21.34	1.22	5.74	6.30	7.55	16.18	0.83	0.07	This Work
CsSnBr_3_	43.89	6.69	5.21	19.09	8.94	23.19	2.135	0.30	This Work
	44.65	3.93	6.57	17.50	10.55			0.08	Cal.^20^
CsSnCl_3_	50.66	8.71	6.01	22.70	10.20	26.61	2.221	0.30	This Work
CsGeI_3_	40.32	8.18	8.87	18.89	11.28	28.22	1.674	0.25	This Work
CsGeBr_3_	48.08	10.82	10.07	23.24	12.92	32.70	1.799	0.27	This Work
	49.52	11.63	11.52	24.26	14.08			0.19	Cal.^20^
CsGeCl_3_	54.93	13.08	11.99	27.03	15.02	38.02	1.800	0.27	This Work

The results of bulk modulus, shear modulus, Young’s modulus, Pugh’s ratio and Poisson ratio of the perovskite compounds were calculated using the second order elastic constants and summarized in Table 1. We also compare our results with available theoretical and experimental results previously reported. The results of bulk modulus for CsPbX_3_ (X = I, Br, Cl) calculated in this work are found to be in reasonable agreement with previous theoretical calculations^21–23^. The only experimental result of elastic moduli for CsPbI_3_ reported by Rakita *et al*.^24^ also agrees well with our simulation. In addition, calculated moduli for CsSnBr_3_ and CsGeBr_3_ match very well with literature^20^. The bulk modulus is an indicator of the stiffness of a volume of material. The calculated values of bulk modulus of the perovskite lie between 6.30 GPa (for CsSnI_3_) and 27.03 GPa (for CsGeCl_3_), indicating that these perovskite compounds are flexible. Because of their flexibility and softness, the metal halide perovskites can easily be developed into thin film and make them more useful in applications such as solar cells. By changing the halogen atom from I to Br then to Cl, the value of bulk modulus is seen to increase. Similarly, the replacement of Pb by Sn and then Sn by Ge increases the bulk modulus of the perovskite compounds except CsSnI_3_ which shows extremely low value of bulk modulus. Similar trend is observed for shear and Young’s modulus with no exception.

**Figure 6:**
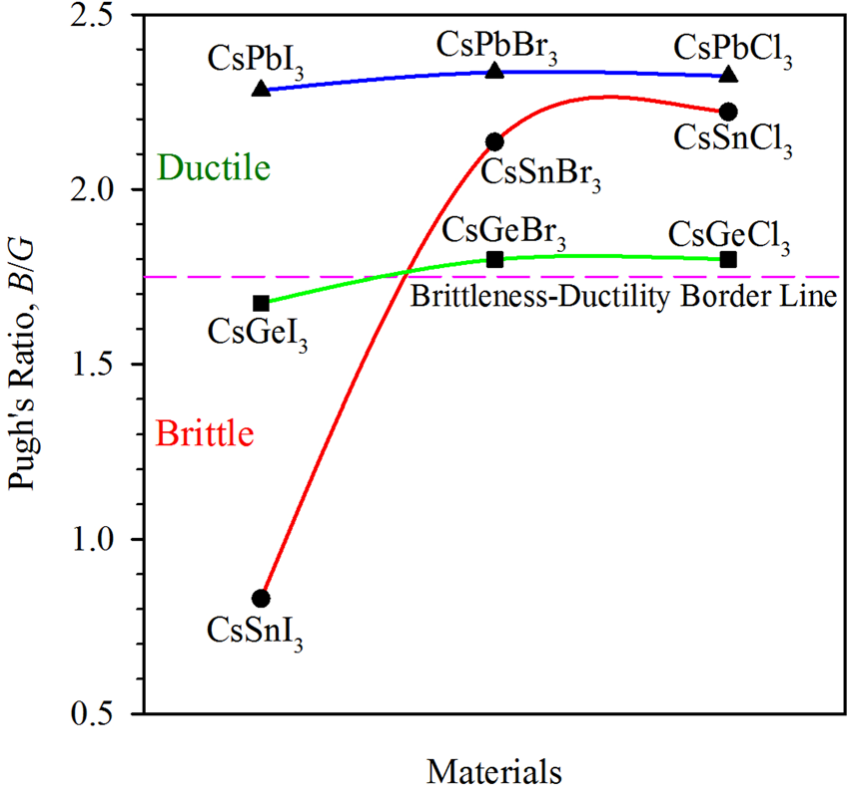
Variation of the Pugh’s ratio of the perovskite with different composition. The pink dashed line separates the ductile materials from brittle.

The ratio of the bulk modulus to shear modulus (*B*/*G*) also known as Pugh’s ratio and the Poisson ratio are considered as a measure to predict the failure mode, i.e., ductility and brittleness of a material^25^. The critical value of Pugh’s ratio to separate the ductile materials from brittle is found to be 1.75, while the critical value of Poisson ratio for the separation of ductile from brittle is ~0.26^25^. If the value of Pugh’s ratio is greater than 1.75 or the value of Poisson ratio is greater than 0.26, then the material is considered as ductile otherwise it is brittle. Also, the higher value of Pugh’s ratio and the Poisson ratio indicate more ductility of a material. According to the criteria, CsSnI_3_ is brittle, CsGeI_3_ is found near brittle-ductile boarder line and the rest of the compounds are ductile in nature. Figure 6 presents the variation of Pugh’s ratio for different compounds. The same characteristics are observed for Poisson ratio (Supplementary Figure 11). The smaller values of Pugh’s ratio and the Poisson ratio of CsSnI_3_ indicate that the material is very brittle and it would not be convenient to handle for applications in a device. The higher ductility is observed in compounds containing Pb. Among them, CsPbBr_3_ shows the highest ductility.

## Lead Free Perovskites

The summary of the key properties of the metal halide perovskites are presented in Table 2. In comparison, Ge based compounds have a higher optical absorption and optical conductivity than Pb containing compounds, which suggests that Ge would be a better replacement of lead.

**Table 2:** Summary of the key properties to predict lead free perovskites.

**CsPbI**_**3**_ Less Optical Absorption Medium Photoconductivity Highly Ductile	**CsPbBr**_**3**_ Less Optical Absorption Less Photoconductivity Highly Ductile	**CsPbCl**_**3**_ Less Optical Absorption Less Photoconductivity Highly Ductile
**CsSnI**_**3**_ Medium Optical Absorption Medium Photoconductivity Highly Brittle	**CsSnBr**_**3**_ Medium Optical Absorption Less Photoconductivity Ductile	**CsSnCl**_**3**_ Medium Optical Absorption Less Photoconductivity Ductile
**CsGeI**_**3**_ High Optical Absorption High Photoconductivity Less Brittle	**CsGeBr**_**3**_ High Optical Absorption Medium Photoconductivity Ductile	**CsGeCl**_**3**_ High Optical Absorption Medium Photoconductivity Ductile

On the other hand, CsSnI_3_ is not suitable to use in any device as it is highly brittle. Furthermore, the Sn based compounds CsSnX_3_ (X = I, Br, Cl) have low optical absorption and conductivity than that of Ge based compounds. Considering all these properties, we can conclude that CsGeI_3_ would be a better lead-free perovskite material for solar cell application as it has highest absorption and photoconductivity in the solar spectrum. However, the problem with CsGeI_3_ is that it is slightly brittle and also having poor film forming ability^10^. Nevertheless the issue of brittleness should be improved by incorporation of Br with CsGeI_3_ in solid solution^26^, and consequently the film forming ability may also be improved.

**Figure 7:**
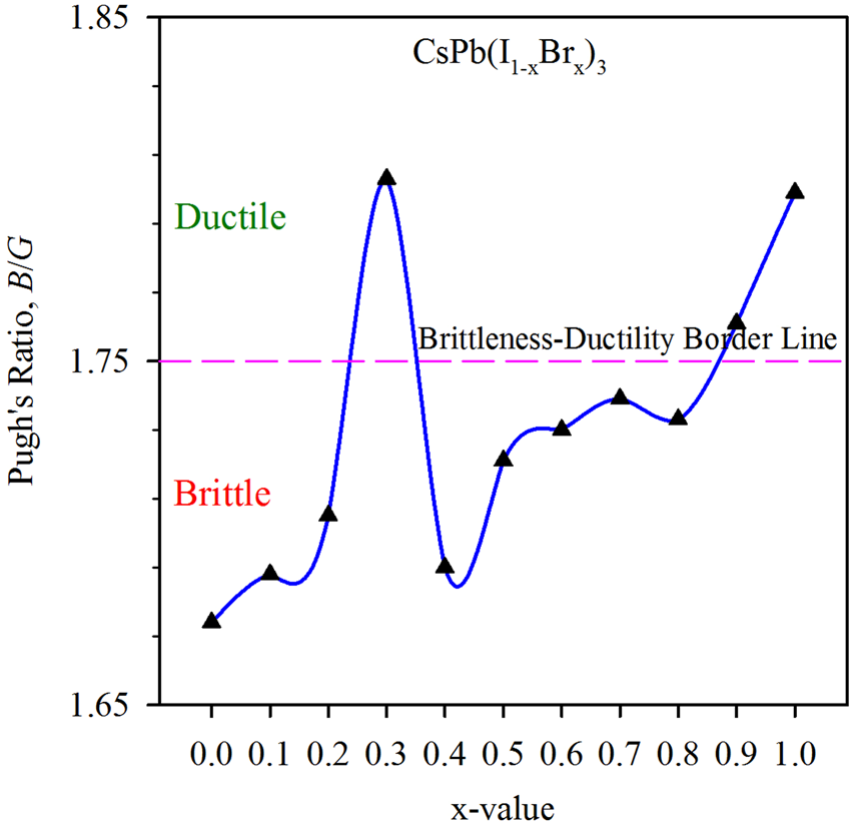
Variation of Pugh’s ratio for different combination of I and Br in CsGe(I_1−x_Br_x_)_3_. CsGe(I_0.7_Br_0.3_)_3_, CsGe(I_0.1_Br_0.9_)_3_ and CsGeBr_3_ are ductile while the others are brittle and the maximum ductility is found in CsGe(I_0.7_Br_0.3_)_3_.

To find a ductile lead free perovskite, the mechanical properties of solid solutions CsGe(I_1−x_Br_x_)_3_ have been calculated and the variation of the Pugh’s ratio for different combinations of I and Br is presented in Fig. 7. According to the results, the maximum ductility is found at *x* = 0.3 in CsGe(I_1−x_Br_x_)_3_. Therefore, CsGe(I_0.7_Br_0.3_)_3_ is more ductile than CsGeI_3_ also the material is found to be mechanically stable and soft (see Supplementary Table 3) and can easily be developed into thin film. The electronic band gap of CsGe(I_0.7_Br_0.3_)_3_ is found as 0.83 eV, also the absorption and the optical conductivity is expected between that of CsGeI_3_ and CsGeBr_3_ as the optical properties is seen to change periodically (Supplementary Figure 10). Therefore CsGe(I_0.7_Br_0.3_)_3_ would be the best Pb-free perovskite for those applications in which ductility is required.

## Conclusion

First-principles DFT calculations have been performed to investigate the structural, electronic, optical and mechanical properties of Pb-free inorganic metal halide cubic perovskites CsBX_3_ (B = Sn, Ge; X = I, Br, Cl) and compared with Pb based compounds. This study suggests that the electronic band gap value is affected due to the replacement of halogen atom while the other properties remain almost same. This is a key feature of the materials to be used in light emitting diodes (LED) as the band gap of these materials can easily be tuned by changing the halogen contents. The investigated perovskite compounds are mechanically stable and can easily be developed into thin films as the materials have low bulk modulus. According to the optical properties analysis, Ge atom appears superior to Pb for the considered inorganic perovskites, as Ge based compounds have higher optical absorption and optical conductivity than the Pb based compounds. By analysing the combined effects of the electronic, optical as well as mechanical properties of CsGeX_3_ (X = I, Br, Cl), it can be concluded that CsGeI_3_ is a better candidate as a Pb- free perovskite for solar cell application. However, CsGeI_3_ is slightly brittle and may not be superior in applications that require ductility. This issue has been resolved by partially replacing iodine with solid solution of Br atom CsGe(I_0.7_Br_0.3_)_3_.

## Computational Methods

The first-principles density functional theory (DFT)^27,28^ calculations have been performed using the plane wave pseudopotential method as implemented in CASTEP^29^ module of Materials Studio 8.0. Vanderbilt type ultrasoft pseudopotential^30^ was used to treat electron ion interaction for all calculations. The exchange-correlation energy was evaluated by using the generalized gradient approximation (GGA) of Perdew-Berke-Ernzerhof (PBE)^31^. To optimize the crystal structure and find the ground state energy the Broyden–Fletcher–Goldfarb–Shanno (BFGS)^32^ minimization technique was used. Elastic constants were calculated by using the finite strain theory^18^ which was implemented within CASTEP module.

We have used the plane-wave cutoff energy as 550 eV through the calculations, and for the sampling of the Brillouin zone the Monkhorst-Pack scheme^33^ *k*-points of 12 × 12 × 12 has been taken. The convergence thresholds of 5 × 10^−6^ eV/atom for the total energy, 0.01 eV/Å for the maximum force, 0.02 GPa for maximum stress, and 5 × 10^−4^ Å for the maximum displacements were used to optimize the geometry of crystal structure.

## Electronic supplementary material

Supplementary Information
