# Supplementary material for: Towards lead-free perovskite photovoltaics and optoelectronics by ab-initio simulations
Source: Sci Rep. 2017 Oct 25;7:14025. doi: 10.1038/s41598-017-13172-y (PMC5656601; doi:10.1038/s41598-017-13172-y)
Supplement: Supplementary file 1 — Supplementary Information [file 41598_2017_13172_MOESM1_ESM.pdf]

Towards lead-free perovskite photovoltaics and optoelectronics by *ab-initio* simulations

Md Roknuzzaman, Kostya (Ken) Ostrikov, Hongxia Wang, Aijun Du, and Tuquabo Tesfamichael*

School of Chemistry, Physics and Mechanical Engineering and Institute of Future Environments, Queensland University of Technology, QLD 4000, Australia

Literature Review

The structural properties of our considered materials have been carried out by several groups either theoretically[1-16] or experimentally[16-23] because this group of compounds are known to the science community for many years. In addition to this, the calculations of the electronic properties of these materials have been done by several groups[5-15,24-27] because of the potentiality of these materials to be used in electronic devices. In contrast, the elastic and details optical properties have been studied less. The bulk modulus of $CsPbX_3$ (X = I, Br, Cl) has been calculated by Chang et al.[5] and Murtaza et al.[6], separately. However, Afsari et al.[7] only calculated the bulk modulus of $CsPbI_3$. The shear modulus, Young's modulus, Pungh's ratio and Poisson's ratio of $CsPbI_3$ are experimentally calculated and reported by Rakita and his co-workers[28]. Moreover, the details elastic properties of $CsPbCl_3$ have been investigated by Ghebouli et al.[8]. In addition to this, the elastic constants as well as the bulk and shear modulus of $CsSnBr_3$ and $CsGeBr_3$ have been studied by Brik[12]. On the other hand, the absorbance and photoluminescence of $CsSnX_3$ (X = I, Br, Cl) have been analysed by Jellicoe et al.[29], experimentally. Also, some theoretical results of the optical properties of these materials have been found in literature. Murtaza and Ahmad[6] have calculated the detail optical properties of $CsPbX_3$ (X = I, Br, Cl) including real and imaginary part of dielectric function, refractive index, extinction coefficient, reflectivity, optical conductivity and optical absorption, whereas Afsari et al.[7] investigated the similar properties for only $CsPbI_3$. In addition to this, the detail optical properties of $CsPbCl_3$ have been investigated by Ghebouli et al.[8]. Also, the dielectric functions of $CsSnBr_3$ and $CsGeBr_3$ have been calculated and analysed by Brik[12] for both the local density approximation (LDA) and generalized gradient approximation (GGA). Moreover, the optical properties of $CsSnX_3$ (X = I, Br, Cl) have been calculated in details by Hayatullah et al.[30].

* Corresponding author.
Email address: t.tesfamichael@qut.edu.au (T. Tesfamichael)

Results with Discussion

Structural Parameters

The calculated equilibrium lattice parameter *a* with available theoretical as well as experimental results and the volume of the optimized cell of our considered nine perovskite compounds are presented in **Supplementary Table 1**. Our calculated values are found in good agreement with the experimental results as well as with the available theoretical results. We have also found that the replacement of halogen atom (X) by a lighter and smaller halogen atom reduces the unit cell volume as well as the lattice parameter. Similarly, the replacement of lead (Pb) by tin (Sn) or germanium causes the reduction of unit cell volume of perovskites. The variation of the unit cell volume due to the replacement of atoms from perovskites is clearly shown in **Supplementary Figure 1**.

Supplementary Table 1. The calculated and the available theoretical and experimental values of lattice parameter *a* (in Å) and the calculated unit cell volume *V* (in Å^3) of perovskites $CsBX_3$ (B = Pb, Sn, Ge; X = I, Br, Cl).

	a			**V**
**Phase**	**This Work**	**Other Works**	**Experimental**	
$CsPbI_3$	6.405	6.13[1], 6.05[5], 6.18[6], 6.40[7]	6.177[17], 6.289[18]	262.7
$CsPbBr_3$	6.013	5.94[2], 5.92[3], 5.74[5], 5.84[6]	5.874[19]	217.4
$CsPbCl_3$	5.743	5.67[2], 5.64[3], 5.49[5], 5.56[6], 5.73[8]	5.605[19]	189.5
$CsSnI_3$	6.260	6.25[2], 5.93[3], 6.15[4], 6.22[9], 6.23[10]	6.219[20]	245.3
$CsSnBr_3$	5.882	5.81[2], 5.57[3], 5.80[9], 5.80[11], 5.80[12]	5.804[21], 5.800[22]	203.5
$CsSnCl_3$	5.613	5.56[9], 5.60[10]	5.560[21]	176.9
$CsGeI_3$	5.978	5.99[16]	5.983[16]	213.6
$CsGeBr_3$	5.587	5.36[12], 5.64[13], 5.69[16]	5.636[16], 5.362[23]	174.4
$CsGeCl_3$	5.314	5.43[13], 5.52[14], 5.24[15], 5.51[16]	5.434[16]	150.1

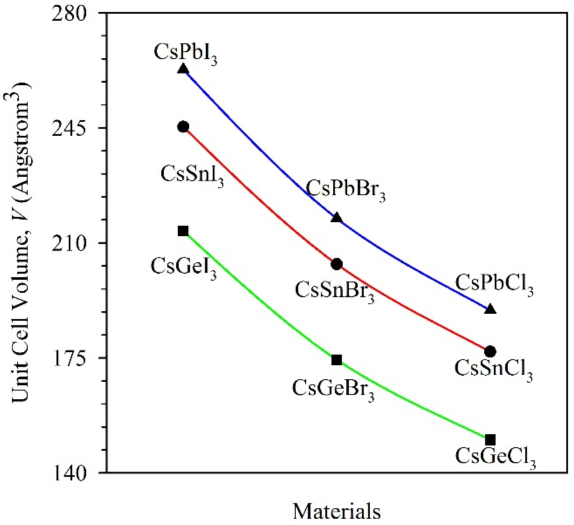

Supplementary Figure 1. Variation of the unit cell volume due to the replacement of atoms by similar atoms.

Electronic Property Analysis

From **Supplementary Table 2**, we have found that the electronic band gaps calculated by Körbel *et al.*[27] using the HSE (Heyd-Scuseria-Ernzerhof)[31] potential are more similar with the experimentally measured optical band gaps. The HSE potential should be better one than other, but it is not the perfect one as diversity has been found for some compounds.

Supplementary Table 2. The calculated electronic band gap using GGA-PBE along with available theoretical results and experimental optical bandgap for perovskites $CsBX_3$ (B = Pb, Sn, Ge; X = I, Br, Cl) in eV.

Phase	This	Other Works	Expt.
$CsPbI_3$	1.479	1.11 (LDA)[5], 1.3[6], 1.56 (PBE)[7], 1.56 (LDA)[24], 0.60 (PAW)[25], 1.51 (PBE)[27], 2.04 (HSE)[27]	1.73[24]
$CsPbBr_3$	1.796	1.12 (LDA)[5], 1.6[6], 2.07 (PBE)[27], 2.42 (HSE)[27]	2.3[32]
$CsPbCl_3$	2.202	1.46 (LDA)[5], 1.8[6], 1.53 (LDA)[8], 2.27 (PP-PW)[8], 2.48 (PBE)[27], 2.95 (HSE)[27]	3.0[32]
$CsSnI_3$	0.406	0.29 (LDA)[9], 1.35 (QSGW)[9], 0.348[10], 0.59 (PBE)[27], 0.85 (HSE)[27]	1.31[22]
$CsSnBr_3$	0.615	0.35 (LDA)[9], 1.69 (QSGW)[9], 1.56 (PBE-GW)[11], 0.35 (PBE)[12], 0.42 (LDA)[26], 0.64 (PBE)[27], 1.11 (HSE)[27]	1.75[22]
$CsSnCl_3$	0.943	0.74 (LDA)[9], 2.99 (QSGW)[9], 0.95[10], 1.00 (PBE) [27], 1.57 (HSE)[27]	2.80[22]
$CsGeI_3$	0.585	1.02[16], 1.17 (PBE)[27], 1.48 (HSE)[27]	1.53[16]
$CsGeBr_3$	0.707	0.55 (PBE)[12], 4.74 (EHTB)[13], 1.49[16], 1.48 (PBE)[27], 1.97 (HSE)[27]	2.32[16]
$CsGeCl_3$	0.978	7.91 (EHTB)[13], 2.11 (PBE)[14], 0.82 (PBE)[15], 2.26[16], 2.17 (PBE)[27], 2.48 (HSE)[27]	3.67[16]

The variation of the optical band gap for different compounds are plotted in **Supplementary Figure 2**. Here, higher band gap is observed for the compounds containing lighter halogen atoms. However, exception is observed in case of replacement of Pb by Sn and Ge. The Germanium (Ge) containing compounds have the higher optical band gaps than that of the Tin (Sn) containing compounds. Also, an exception is observed for $CsGeCl_3$, a maximum optical band gap is observed for this material.

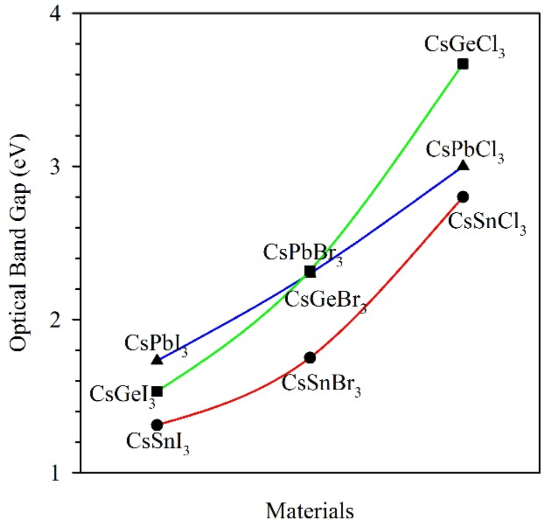

Supplementary Figure 2. Variation of optical band gap due to the replacement of atoms by similar atoms. The data of optical band gap are taken from references[24], [32], [22] and [16].

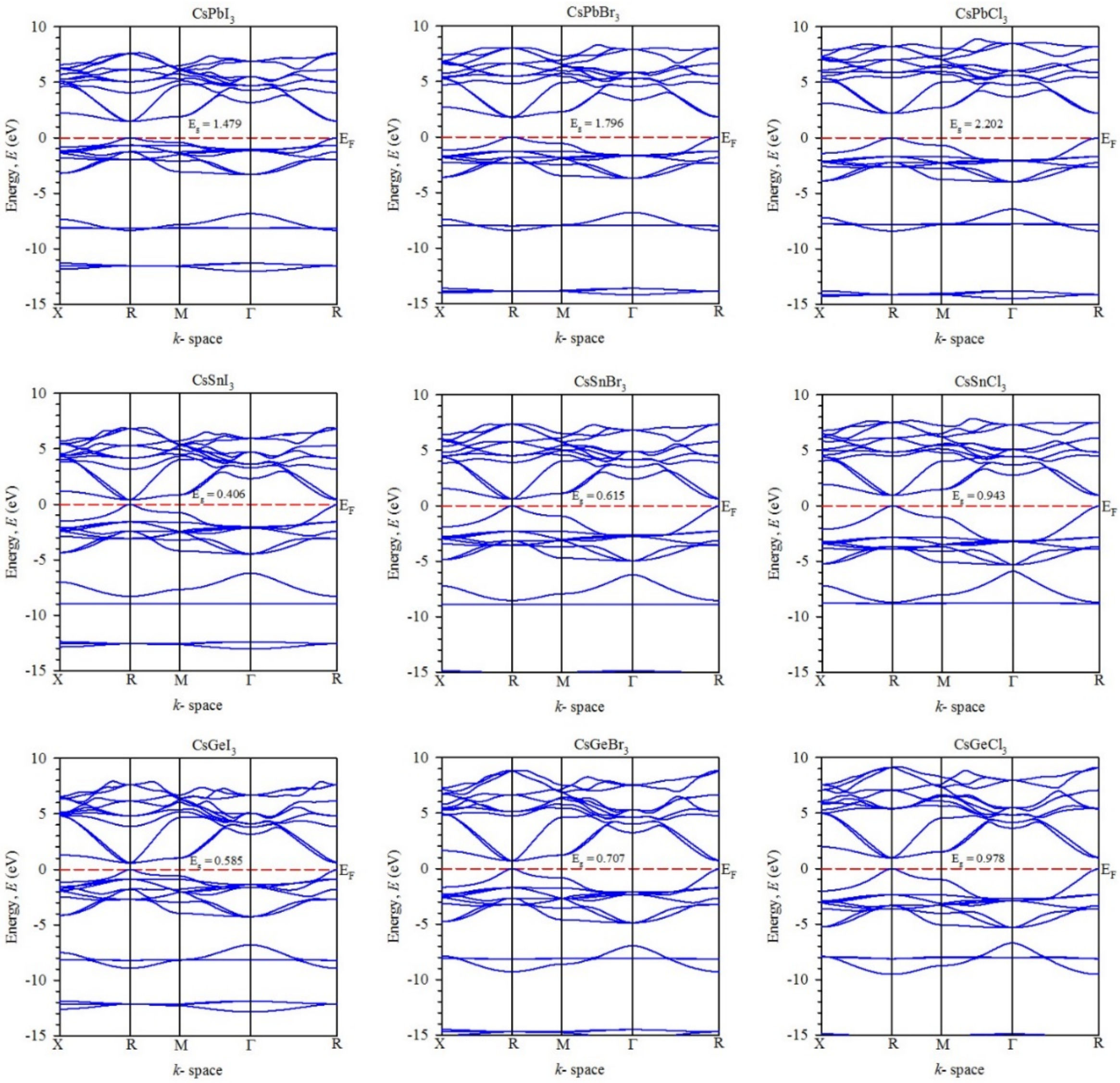

Supplementary Figure 3. Electronic band structure of perovskites $CsBX_3$ (B = Pb, Sn, Ge; X = I, Br, Cl).

The calculated electronic band structure of $CsBX_3$ (B = Pb, Sn, Ge; X = I, Br, Cl) perovskites along the high symmetry direction of the first Brillioun zone is shown in **Supplementary Figure 3**. Our calculations reveal that the considered compounds are direct band gap semiconductors having minimum band gap at the *R* point of the Brilloun zone. The band gap value is seen to increase by replaceing I with Br as well as Br with Cl. But, from the band structure figure (**Supplementary Figure 3**) it is observed that the pattern of the band structure is almost not affected by replacement of a halogen atom with other halogen atoms. Due to this characteristics, metal halide perovskites should be used in light emitting diodes (LED) as the band gap of these materials can easily be tuned by changing the halogen contents while the other properties remain almost same. On the other hand, a slight change in band structure is observed during the replacement of Pb by Sn or Ge.

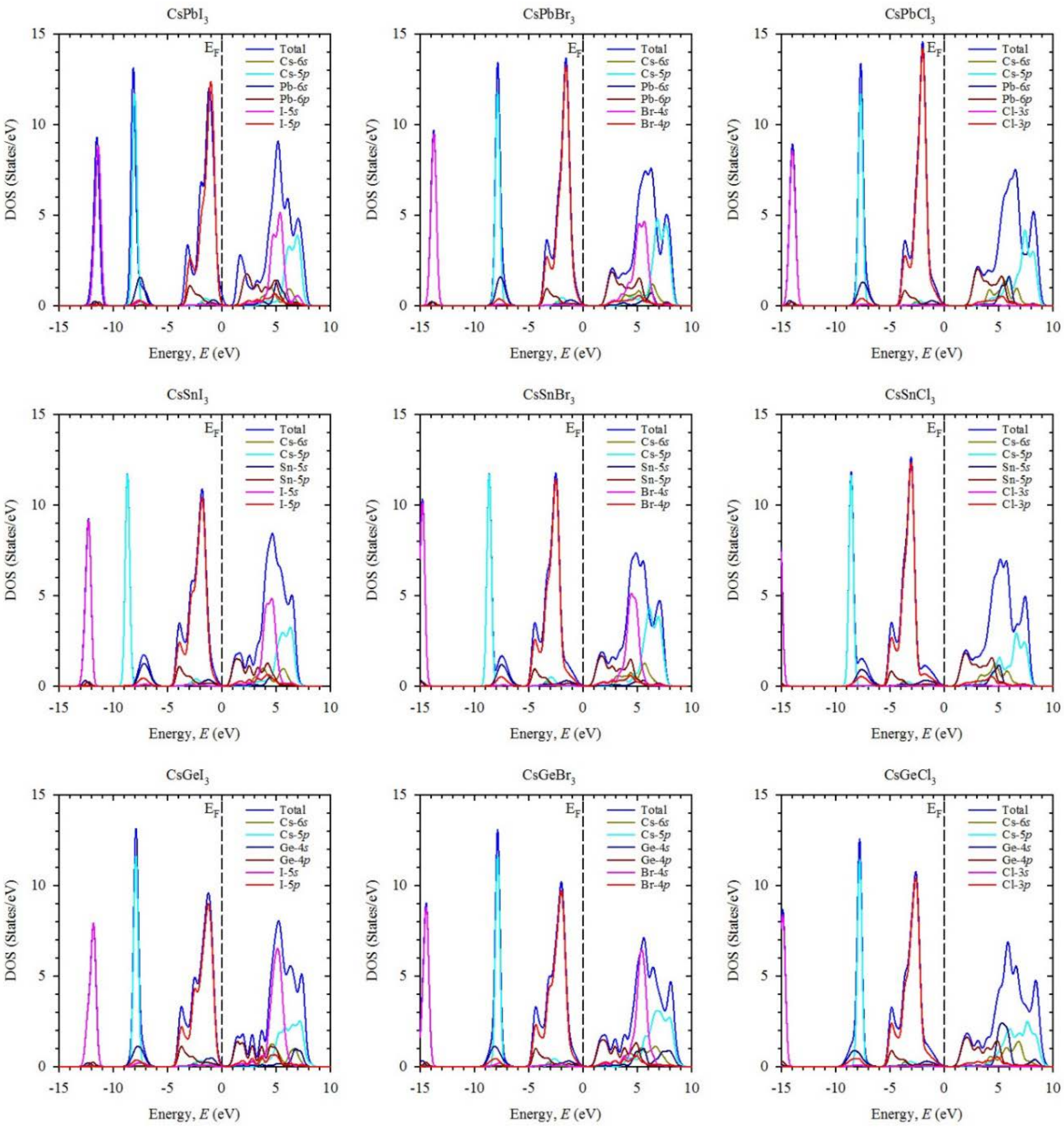

Supplementary Figure 4. Total and partial densities of states of perovskites $CsBX_3$ (B = Pb, Sn, Ge; X = I, Br, Cl). The Fermi level E_F is set at 0 eV.

The total and partial densities of states of our considered nine perovskite compounds is shown in **Supplementary Figure 4**. The total DOS below the fermi level is seen to increase for the compounds from left to right in the figure. On the other hand, this value is reduced for the compounds from top to bottom in **Supplementary Figure 4**. It suggests that the replacement of I by Br and Cl increase the number of electronic states of valence band just below the fermi level. But the opposite trend is observed during the replacement of Pb by Sn or Ge. Moreover, the total DOS below the Fermi level mainly comes from the *p* orbital of halogen atoms. In addition to this the total DOS above the Fermi level is mainly contributed by *p* orbital of B atoms as by Pb-6*p*, Sn-5*p* and Ge-4*p*.

Optical Functions

The calculated real and imaginary part of the dielectric functions of nine metal halide perovskites $CsBX_3$ (B = Pb, Sn, Ge; X = I, Br, Cl) are shown in **Supplementary Figure 5** and **Supplementary Figure 6**, respectively. The response of a material to incident electromagnetic wave can be characterised by dielectric function. The real part of dielectric function of $CsBI_3$ (B = Pb, Sn, Ge) have two peaks and a shoulder. However, the shoulder of the real part of the dielectric function become the third peak during the replacement of I by Br or Cl. A comparative study reveals that the Ge containing compounds have greater magnitude at low energy region but it fluctuate more while for Pb containing compounds the fluctuation is less than others. Also, the magnitude of the real part of the dielectric function is seen to reduce when I is replaced by Br and Br is replaced by Cl.

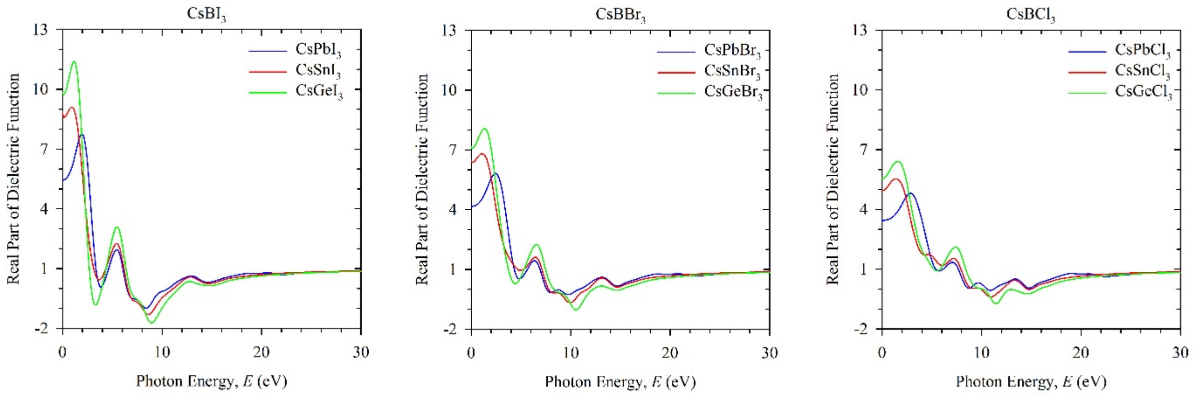

Supplementary Figure 5. Calculated real part of dielectric function of perovskites $CsBX_3$ (B = Pb, Sn, Ge; X = I, Br, Cl) as a function of incident photon energy.

A similar trend is observed for the magnitude of the imaginary part of the dielectric function (**Supplementary Figure 6**) occupying three peaks and one shoulder for the considered compounds. The imaginary part of the dielectric function tends to zero at high energy (more than 16 eV) region. On the other hand, the real part of the dielectric function become unity at high energy implies that the materials become transparent with very little absorption for high energy incident photon.

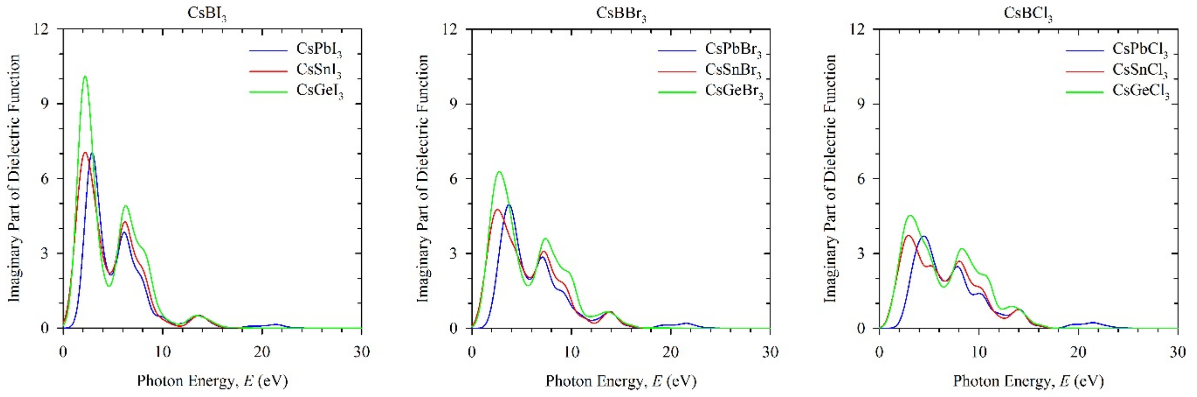

Supplementary Figure 6. Calculated imaginary part of dielectric function of perovskites $CsBX_3$ (B = Pb, Sn, Ge; X = I, Br, Cl) as a function of incident photon energy.

The reflectivity is another important parameter for the device applications of a material. The considered materials show a low reflectivity in the IR-Visible-UV region of the spectra (**Supplementary Figure 7**). This low reflectivity suggests that the materials have high absorptivity and transmitivity. However, our study of the dielectric function suggest that the materials have high transmitivity at high energy region. Therefore, the investigation of the reflectivity and dielectric function also evident that the considered compounds have high absorption at low energy (IR-Visible-UV) region of the spectrum.

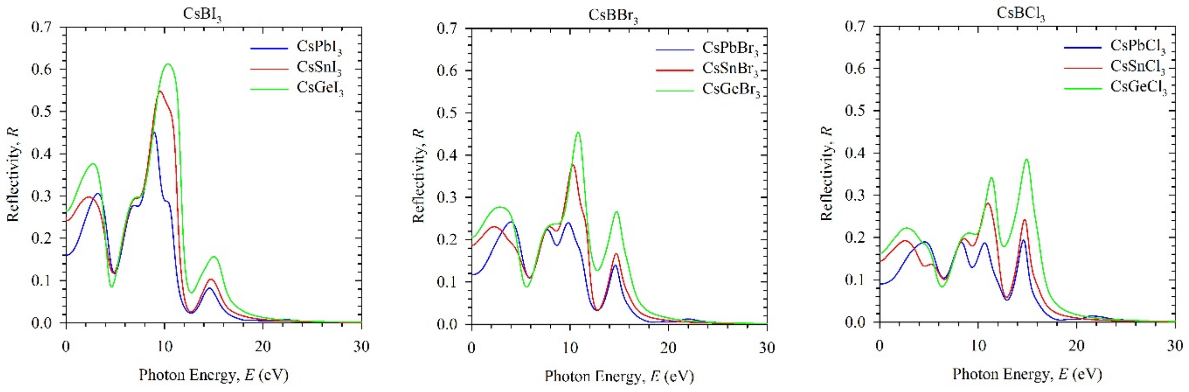

Supplementary Figure 7. Calculated reflectivity of perovskites $CsBX_3$ (B = Pb, Sn, Ge; X = I, Br, Cl) as a function of incident photon energy.

The knowledge of refractive index is important for the applications of a material in optical devices like solar cell, photodetector, waveguide and photonic crystal[6]. The overall features of the refractive index for the considered nine perovskites are qualitatively same in the entire energy range except some variation in heights and positions of peaks (**Supplementary Figure 8**). However, the value of the static refractive index *n*(0) is different for different compounds. The maximum value of *n*(0) is found as 3.15 for $CsGeI_3$ while the minimum value if found for $CsPbCl_3$ as 1.85.

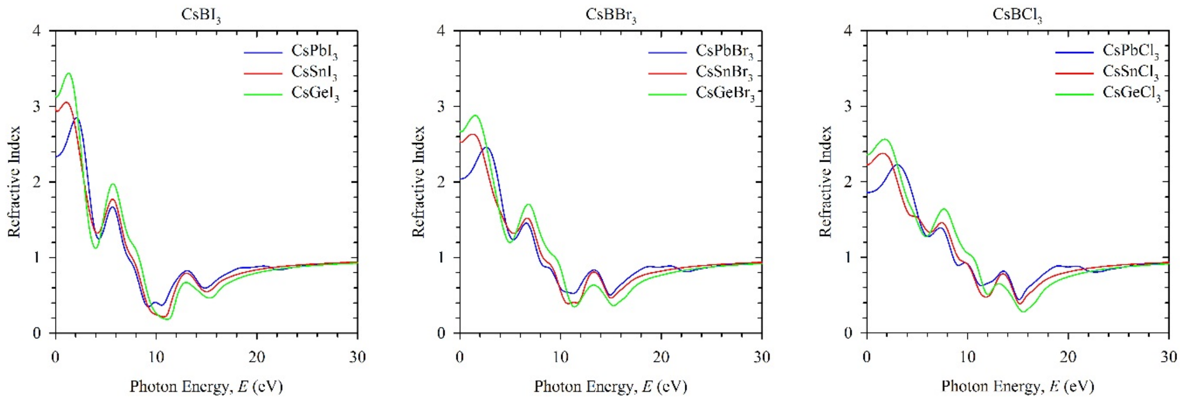

Supplementary Figure 8. Calculated refractive index of perovskites $CsBX_3$ (B = Pb, Sn, Ge; X = I, Br, Cl) as a function of incident photon energy.

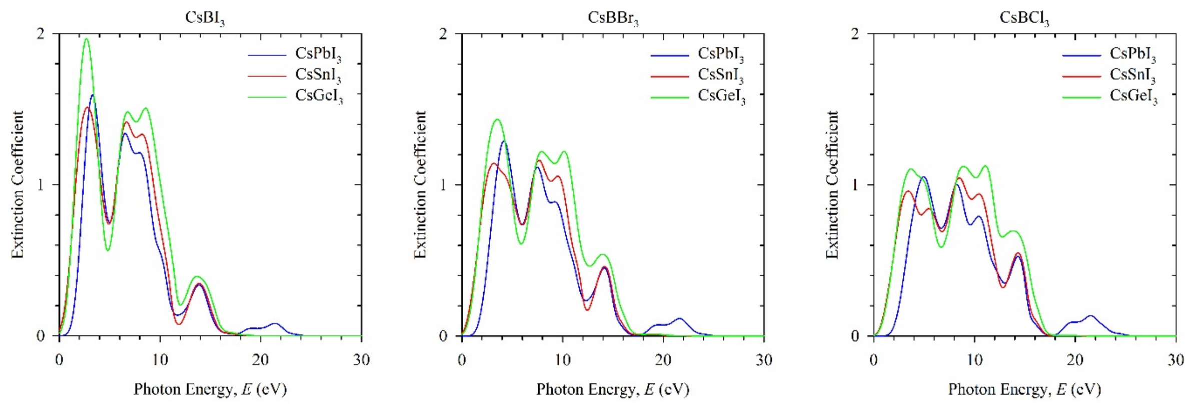

Supplementary Figure 9. Calculated extinction coefficient of perovskites $CsBX_3$ (B = Pb, Sn, Ge; X = I, Br, Cl) as a function of incident photon energy.

The calculated extinction coefficient of the considered perovskites $CsBX_3$ (B = Pb, Sn, Ge; X = I, Br, Cl) is shown in **Supplementary Figure 9**. Similar to the absorption and optical conductivity, the extinction coefficient of Ge based compounds have higher values than that of Pb and Sn based compounds.

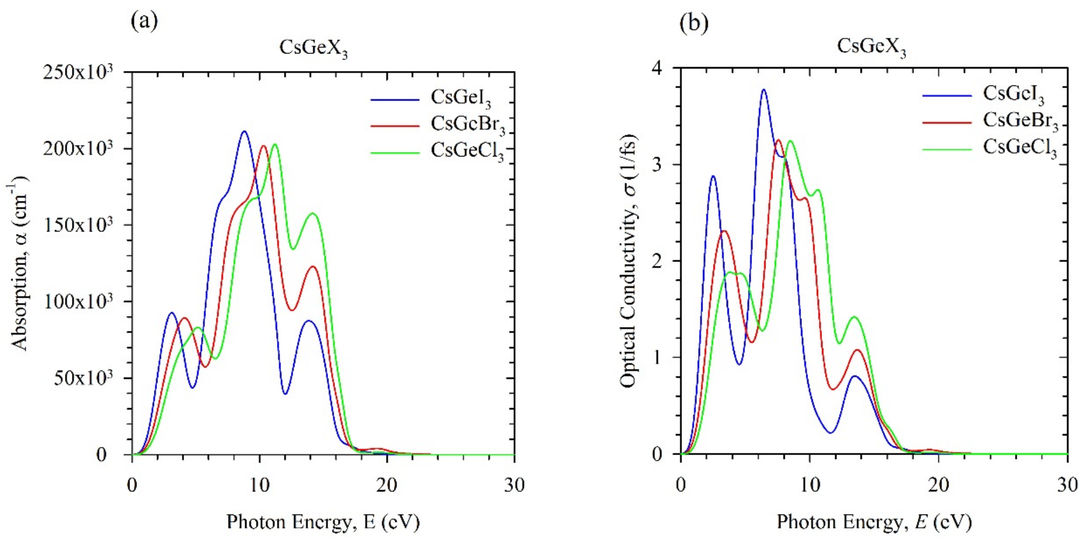

Supplementary Figure 10. Periodic change of optical properties due to replacement of halogen atoms. (a) Calculated optical absorption of perovskites $CsGeX_3$ (X = I, Br, Cl) as a function of incident photon energies. (b) Calculated optical conductivity of perovskites $CsGeX_3$ (X = I, Br, Cl) as a function of incident photon energies.

Elastic Properties

The variation of the Poisson ratio for the considered nine metal halide perovskites is plotted in **Supplementary Figure 11**. The variation of the Poisson ratio is quite identical with the variation of Pugh's ratio. From **Supplementary Figure 11**, it is clearly evident that $CsSnI_3$ is quite brittle and $CsGeI_3$ is less brittle while the other seven compounds are found to be ductile in nature.

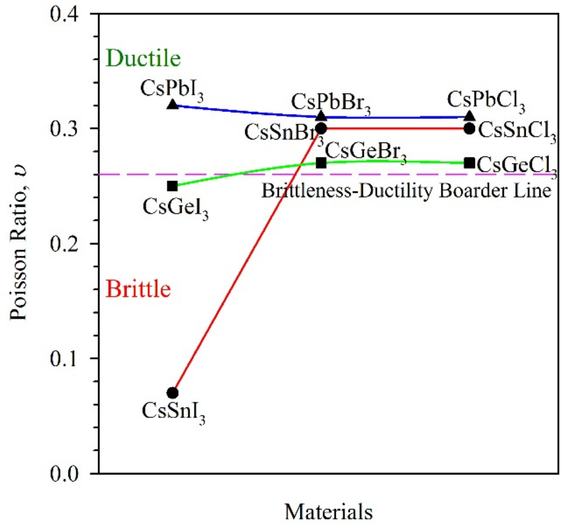

Supplementary Figure 11. Change in Poisson ratio due to the replacement of atoms by similar atoms.

The detail mechanical properties of the considered solid solutions $CsGe(I_{1-x}Br_x)_3$ are presented in **Supplementary Table 3**. According to the Born stability criteria[33], it is clearly observed that the considered solid solutions are mechanically stable. Also, the small value of the bulk modulus suggests that the materials are soft and can easily be converted into thin film which is important for solar cell applications.

Supplementary Table 3. The calculated elastic constants C_{ij} (in GPa), bulk modulus B (in GPa), shear modulus G (in GPa), Young's modulus Y (in GPa), Pugh's ratio B/G and Poisson ratio υ of perovskites solid solutions $CsGe(I_{1-x}Br_x)_3$.

Phase	C_{11}	C_{12}	C_{44}	B	G	Y	B/G	υ
$CsGeI_3$	40.32	8.18	8.87	18.89	11.28	28.22	1.674	0.251
$CsPb(I_{0.9}Br_{0.1})_3$	39.39	7.65	8.31	18.23	10.80	27.06	1.688	0.253
$CsPb(I_{0.8}Br_{0.2})_3$	41.24	7.86	8.45	18.98	11.14	27.95	1.705	0.254
$CsPb(I_{0.7}Br_{0.3})_3$	45.38	10.36	9.58	22.04	12.22	30.94	1.803	0.266
$CsPb(I_{0.6}Br_{0.4})_3$	42.64	8.91	9.43	20.15	11.93	29.89	1.690	0.253
$CsPb(I_{0.5}Br_{0.5})_3$	43.21	8.89	9.16	20.33	11.81	29.68	1.721	0.257
$CsPb(I_{0.4}Br_{0.6})_3$	45.08	9.39	9.56	21.29	12.31	30.96	1.730	0.258
$CsPb(I_{0.3}Br_{0.7})_3$	44.76	9.47	9.51	21.24	12.21	30.74	1.739	0.259
$CsPb(I_{0.2}Br_{0.8})_3$	45.85	9.65	9.76	21.71	12.53	31.53	1.733	0.258
$CsPb(I_{0.1}Br_{0.9})_3$	46.77	10.11	9.87	22.33	12.68	31.99	1.761	0.261
$CsGeBr_3$	48.08	10.82	10.07	23.24	12.92	32.70	1.799	0.265

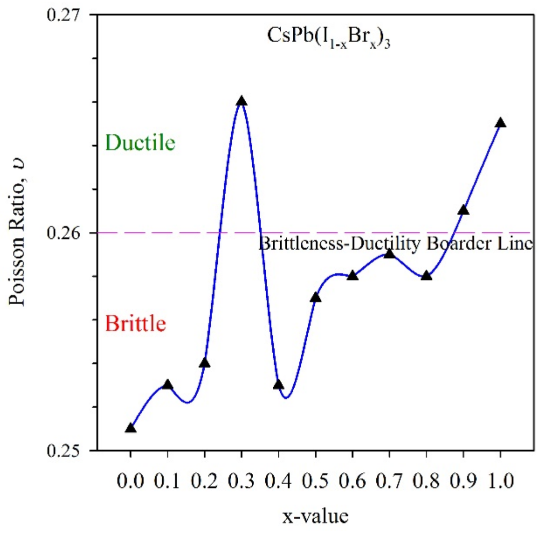

Supplementary Figure 12. Change in Poisson ratio for the solid solutions $CsGe(I_{1-x}Br_x)_3$, where x = 0-1 at the increment of 0.1.

The variation of the Poisson ratio for the different combinations of I and Br in the solid solutions $CsGe(I_{1-x}Br_x)_3$ is shown in **Supplementary Figure 12**. From the figure it is clearly evident that the solid solutions $CsGe(I_{0.7}Br_{0.7})_3$ and $CsGe(I_{0.1}Br_{0.9})_3$ and the compound $CsGeBr_3$ are found to be ductile while the other eight combinations are brittle in nature. Also, the maximum ductility is observed for the solid solution $CsGe(I_{0.7}Br_{0.7})_3$. This characteristic is quite identical with the result of Pugh's ratio.

Theoretical Calculations

Optimized Structure: The first-principles calculations have been carried out to obtain the optimized structure of $CsBX_3$ (B = Pb, Sn, Ge; X = I, Br, Cl). The calculations based on density functional theory (DFT) were performed using CASTEP[34] module of Materials Studio simulation package.

Optical Properties: The optical properties of crystalline solids can be described by the complex dielectric function, $\varepsilon(\omega) = \varepsilon_1(\omega) + i\varepsilon_2(\omega)$; where $\varepsilon_1(\omega)$ and $\varepsilon_2(\omega)$ are the real and imaginary part of the dielectric function, respectively. The imaginary part of the dielectric function is essential to calculate other optical constants[35]. The imaginary part of the dielectric function can be calculated from the momentum matrix elements related to the occupied and unoccupied wave functions in accordance with the selection rules[36] and can be represented as:

$$\varepsilon_2(\omega) = \frac{2e^2\pi}{\Omega\varepsilon_0}\sum_{k,v,c}|\langle\psi_k^c|\boldsymbol{u}\cdot\boldsymbol{r}|\psi_k^v\rangle|^2\,\delta(E_k^c - E_k^v - E)$$

In the above formula, ω is the phonon frequency, e is the electronic charge, Ω is the volume of a unit cell, $\boldsymbol{u}$ is the unit vector along the polarization of the incident electric field and ψ_k^c and ψ_k^v are respective wave functions for conduction and valence band electrons at a particular k. Using the Kramers-Kronig relations, the real part of the dielectric function can be derived from the imaginary part. Other remaining optical functions, such as the refractive index $n(\omega)$, extinction coefficient $k(\omega)$, absorption coefficient $\alpha(\omega)$, energy-loss function $L(\omega)$, optical conductivity $\sigma(\omega)$ and reflectivity $R(\omega)$, can be obtained from the both real and imaginary parts of the dielectric functions $\varepsilon_1(\omega)$ and $\varepsilon_2(\omega)$ as described in the literature[37].

Mechanical Properties: Our considered CASTEP module uses the finite strain theory[38] to calculate the elastic constants of a range of materials including semiconductors. In this approach, the elastic constants are calculated from first-principles calculations by applying a set of given homogeneous deformations with a finite value and calculating the resulting stress with respect to optimizing the internal atomic coordinates, i.e., optimizing geometry with fixed cell parameters. The elastic constants are then the proportionality constants relating the applied strain to the computed stress, $\sigma_i = C_{ij}\varepsilon_j$. The cubic perovskites have three independent elastic constants, such as C_{11}, C_{12}, and C_{44}. These constants are determined with the 'stress-strain' method[39] as implemented in the CASTEP module. The polycrystalline elastic moduli such as bulk modulus, shear modulus are then derived from the single crystal elastic constants applying the Hill approximations[40]. Also, the Young's modulus and the Poisson ratio can be calculated from the values of bulk modulus and shear modulus using the well-known formula, $Y = (9BG)/(3B + G)$ and $\upsilon = (3B - 2G)/(6B + 2G)$, respectively.

References

1 Da Silva, E. L., Skelton, J. M., Parker, S. C. & Walsh, A. Phase stability and transformations in the halide perovskite $CsSnI_3$. *Physical Review B* **91**, 144107 (2015).

2 Jiang, L. *et al.* Prediction of lattice constant in cubic perovskites. *Journal of Physics and Chemistry of Solids* **67**, 1531-1536 (2006).

3 Moreira, R. L. & Dias, A. Comment on "Prediction of lattice constant in cubic perovskites". *Journal of Physics and Chemistry of Solids* **68**, 1617-1622 (2007).

4 Yu, C., Ren, Y., Chen, Z. & Shum, K. First-principles study of structural phase transitions in $CsSnI_3$. *Journal of Applied Physics* **114**, 163505 (2013).

5 Chang, Y., Park, C. & Matsuishi, K. First-principles study of the structural and the electronic properties of the lead-halide-based inorganic-organic perovskites $(CH_3NH_3)PbX_3$ and $CsPbX_3$ (X= Cl, Br, I). *Journal-Korean Physical Society* **44**, 889-893 (2004).

6 Murtaza, G. & Ahmad, I. First principle study of the structural and optoelectronic properties of cubic perovskites $CsPbM_3$ (M= Cl, Br, I). *Physica B: Condensed Matter* **406**, 3222-3229 (2011).

7 Afsari, M., Boochani, A. & Hantezadeh, M. Electronic, optical and elastic properties of cubic perovskite $CsPbI_3$: Using first principles study. *Optik - International Journal for Light and Electron Optics* **127**, 11433-11443 (2016).

8 Ghebouli, M., Ghebouli, B. & Fatmi, M. First-principles calculations on structural, elastic, electronic, optical and thermal properties of $CsPbCl_3$ perovskite. *Physica B: Condensed Matter* **406**, 1837-1843 (2011).

9 Huang, L.-y. & Lambrecht, W. R. Electronic band structure, phonons, and exciton binding energies of halide perovskites $CsSnCl_3$, $CsSnBr_3$, and $CsSnI_3$. *Physical Review B* **88**, 165203 (2013).

10 Borriello, I., Cantele, G. & Ninno, D. Ab initio investigation of hybrid organic-inorganic perovskites based on tin halides. *Physical Review B* **77**, 235214 (2008).

11 Pramchu, S., Laosiritaworn, Y. & Jaroenjittichai, A. Electronic properties of surface/bulk iodine defects of $CsSnBr_3$ perovskite. *Surface and Coatings Technology* **306**, 159-163 (2016).

12 Brik, M. Comparative first-principles calculations of electronic, optical and elastic anisotropy properties of $CsXBr_3$ (X= Ca, Ge, Sn) crystals. *Solid State Communications* **151**, 1733-1738 (2011).

13 Seo, D.-K., Gupta, N., Whangbo, M.-H., Hillebrecht, H. & Thiele, G. Pressure-induced changes in the structure and band gap of $CsGeX_3$ (X= Cl, Br) studied by electronic band structure calculations. *Inorganic chemistry* **37**, 407-410 (1998).

14 Huang, D. *et al.* First-principles prediction of a promising p-type transparent conductive material $CsGeCl_3$. *Applied Physics Express* **7**, 041201 (2014).

15 Erdinc, B. *et al.* Ab-initio study of $CsGeCl_3$ compound in paraelectric and ferroelectric phases. *Ferroelectrics* **494**, 138-149 (2016).

16 Tang, L.-C., Chang, Y.-C., Huang, J.-Y., Lee, M.-H. & Chang, C.-S. First principles calculations of linear and second-order optical responses in rhombohedrally distorted perovskite ternary halides, $CsGeX_3$ (X= Cl, Br, and I). *Japanese Journal of Applied Physics* **48**, 112402 (2009).

17 Eperon, G. E. *et al.* Inorganic caesium lead iodide perovskite solar cells. *Journal of Materials Chemistry A* **3**, 19688-19695 (2015).

18 Trots, D. & Myagkota, S. High-temperature structural evolution of caesium and rubidium triiodoplumbates. *Journal of Physics and Chemistry of Solids* **69**, 2520-2526 (2008).

19 Rohrer, G. S. *Structure and bonding in crystalline materials.* (Cambridge University Press, 2001).

20 Yamada, K. *et al.* Structural phase transitions of the polymorphs of $CsSnI_3$ by means of rietveld analysis of the X-ray diffraction. *Chemistry Letters* **20**, 801-804 (1991).

21 Barrett, J., Bird, S., Donaldson, J. & Silver, J. The Mössbauer effect in tin (II) compounds. Part XI. The spectra of cubic trihalogenostannates (II). *Journal of the Chemical Society A: Inorganic, Physical, Theoretical*, 3105-3108 (1971).

22 Peedikakkandy, L. & Bhargava, P. Composition dependent optical, structural and photoluminescence characteristics of cesium tin halide perovskites. *RSC Advances* **6**, 19857-19860 (2016).

23 Lin, Z.-G., Tang, L.-C. & Chou, C.-P. Characterization and properties of infrared NLO crystals: $AGeX_3$ (A= Rb, Cs; X= Cl, Br). *Journal of Crystal Growth* **310**, 3224-3229 (2008).

24 Filip, M. R., Eperon, G. E., Snaith, H. J. & Giustino, F. Steric engineering of metal-halide perovskites with tunable optical band gaps. *Nature communications* **5** (2014).

25 Brgoch, J., Lehner, A. J., Chabinyc, M. & Seshadri, R. Ab initio calculations of band gaps and absolute band positions of polymorphs of $RbPbI_3$ and $CsPbI_3$: Implications for main-group halide perovskite photovoltaics. *The Journal of Physical Chemistry C* **118**, 27721-27727 (2014).

26 Zheng, J. C., Huan, C., Wee, A. & Kuok, M. Electronic Properties of $CsSnBr_3$: Studies by Experiment and Theory. *Surface and interface analysis* **28**, 81-83 (1999).

27 Körbel, S., Marques, M. A. & Botti, S. Stability and electronic properties of new inorganic perovskites from high-throughput ab initio calculations. *Journal of Materials Chemistry C* **4**, 3157-3167 (2016).

28 Rakita, Y., Cohen, S. R., Kedem, N. K., Hodes, G. & Cahen, D. Mechanical properties of $APbX_3$ (A= Cs or CH_3 NH_3; X= I or Br) perovskite single crystals. *MRS Communications* **5**, 623-629 (2015).

29 Jellicoe, T. C. *et al.* Synthesis and optical properties of lead-free cesium tin halide perovskite nanocrystals. *Journal of the American Chemical Society* **138**, 2941-2944 (2016).

30 Ayatullahah, H. *et al.* Physical properties of $CsSnM_3$ (M= Cl, Br, I): A first principle study. *Acta Phys. Polym* **124**, 102-107 (2013).

31 Heyd, J., Scuseria, G. E. & Ernzerhof, M. Hybrid functionals based on a screened Coulomb potential. *The Journal of Chemical Physics* **118**, 8207-8215 (2003).

32 Heidrich, K. *et al.* Electronic structure, photoemission spectra, and vacuum-ultraviolet optical spectra of $CsPbCl_3$ and $CsPbBr_3$. *Physical Review B* **24**, 5642 (1981).

33 Born, M. On the stability of crystal lattices. I. *Mathematical Proceedings of the Cambridge Philosophical Society* **36**, 160-172 (2008).

34 Clark, S. J. *et al.* First principles methods using CASTEP. *Zeitschrift für Kristallographie-Crystalline Materials* **220**, 567-570 (2005).

35 Roknuzzaman, M. *et al.* First hafnium-based MAX phase in the 312 family, Hf_3AlC2: A first-principles study. *Journal of Alloys and Compounds*, doi:http://dx.doi.org/10.1016/j.jallcom.2017.08.151 (2017).

36 Harris, D. C. & Bertolucci, M. D. *Symmetry and spectroscopy: an introduction to vibrational and electronic spectroscopy*. (Courier Corporation, 1978).

37 Hadi, M. A., Vovk, R. V. & Chroneos, A. Physical properties of the recently discovered $Zr_2(Al_{1-x}Bi_x)C$ MAX phases. *Journal of Materials Science: Materials in Electronics* **27**, 11925-11933 (2016).

38 Murnaghan, F. D. Finite deformations of an elastic solid. *American Journal of Mathematics* **59**, 235-260 (1937).

39 Le Page, Y. & Saxe, P. Symmetry-general least-squares extraction of elastic data for strained materials from ab initio calculations of stress. *Physical Review B* **65**, 104104 (2002).

40 Hill, R. The Elastic Behaviour of a Crystalline Aggregate. *Proceedings of the Physical Society. Section A* **65**, 349 (1952).
